# Synthesis and Characterization of a High Flux Nanocellulose–Cellulose Acetate Nanocomposite Membrane

**DOI:** 10.3390/membranes9060070

**Published:** 2019-06-06

**Authors:** Nancy Li, Jackie Zheng, Pejman Hadi, Mengying Yang, Xiangyu Huang, Hongyang Ma, Harold W. Walker, Benjamin S. Hsiao

**Affiliations:** 1 Department of Chemistry, Stony Brook University, Stony Brook, NY 11794, USA; nancy.li@stonybrook.edu (N.L.); jackie.zheng@stonybrook.edu (J.Z.); mengying.yang@stonybrook.edu (M.Y.); xiangyu.huang@stonybrook.edu (X.H.); hongyang.ma@stonybrook.edu (H.M.); 2 New York State Center for Clean Water Technology, Stony Brook University, Stony Brook, NY 11794, USA; 3 Department of Civil and Environmental Engineering, Worcester Polytechnic Institute, Worcester, MA 01609, USA

**Keywords:** membrane flux, nanocomposite, nanocellulose, mixed matrix membranes, hydrophilic surface, electrostatic repulsion, protein rejection

## Abstract

Despite the advantages of membrane processes, their high energy requirement remains a major challenge. Fabrication of nanocomposite membranes by incorporating various nanomaterials in the polymer matrix has shown promise for enhancing membrane flux. In this study, we embed functionalized cellulose nanofibers (CNFs) with high aspect ratios in the polymer matrix to create hydrophilic nanochannels that reduce membrane resistance and facilitate the facile transport of water molecules through the membrane. The results showed that the incorporation of 0.1 wt % CNF into the polymer matrix did not change the membrane flux (~15 $L\cdot m^{-2}\cdot h^{-1}$) and Bovine Serum Albumin (BSA) Fraction V rejection, while increasing the CNF content to 0.3 wt % significantly enhanced the flux by seven times to ~100 $L\cdot m^{-2}\cdot h^{-1}$, but the rejection was decreased to 60–70%. Such a change in membrane performance was due to the formation of hydrophilic nanochannels by the incorporation of CNF (corroborated by the SEM images), decreasing the membrane resistance, and thus enhancing the flux. When the concentration of the CNF in the membrane matrix was further increased to 0.6 wt %, no further increase in the membrane flux was observed, however, the BSA rejection was found to increase to 85%. Such an increase in the rejection was related to the electrostatic repulsion between the negatively-charged CNF-loaded nanochannels and the BSA, as demonstrated by zeta potential measurements. SEM images showed the bridging effect of the CNF in the nanochannels with high CNF contents.

## 1. Introduction

Despite the unique advantages of membranes for water purification, the high energy demand of the separation process remains a challenge. Relatively high pressures are required to generate sufficient flux through conventional polymeric membranes which are hydrophobic in nature and have low affinity for water molecules [1]. Many attempts have been made to render membranes hydrophilic [2,3]. For example, Zhang et al. blended hydrophilic polyvinyl alcohol (PVA) polymer with hydrophobic polyvinylidene fluoride (PVDF) to increase the hydrophilicity of a casted membrane [4]. Zeng et al. added dopamine-modified halloysite nanotubes into the polymer matrix for enhanced hydrophilicity [5]. Polymer grafting and copolymerization to induce hydrophilicity to polymers are also well-investigated approaches [6,7,8,9,10]. In addition to challenges related to the hydrophobicity of the membranes, their porosity, mostly prepared by nonsolvent-induced phase separation, is relatively low due to the instantaneous solvent-nonsolvent exchange and fast polymer precipitation. These two intrinsic membrane characteristics – comparatively low hydrophilicity and low porosity – lead to low water flux at a given applied pressure.

Extensive past attempts to enhance the water flux of membranes by incorporating nanoparticles [11,12,13,14], nanotubes [15,16,17,18,19], or nanosheets [20,21,22] in the membrane polymer matrix have been successful only to a limited extent. Carbon nanotubes (CNTs) belong to a prototypical class of nanomaterials that have attracted particular attention due to their high surface area and aspect ratio [19]. CNTs create a graphitic nanochannel structure that transports water molecules through aligned CNT membranes in an impermeable matrix [23,24]. The approaches for the fabrication of these highly aligned CNT-polymer nanocomposites are sophisticated and tedious, and hence, impractical for water purification purposes [25]. To overcome this challenge, some researchers have attempted to integrate CNTs as “additives” into the permeable membrane matrix, but CNT incorporation as a filler results in random CNT orientation, losing its nanochannel alignment [26]. In addition, the hydrophobic nature of the CNTs necessitates its functionalization in order to enhance the interfacial adhesion between the two phases (i.e., CNT and polymer) [27,28,29].

Cellulose nanofiber (CNF) is another class of nanofibrous material that is gaining increasing attention. They have a high aspect ratio and surface area and also remarkable mechanical strength, similar to CNTs [30]. CNF can be chemically obtained by surface modification of cellulose fibrils typically by TEMPO-mediated oxidation [31,32], that introduces abundant carboxylate and aldehyde functional groups to the fiber surface, imparting a highly negative surface charge to the fibers and hence, facilitating the homogeneous distribution of the produced nanofibers in suspension form [33]. On the basis of this oxidation mechanism, CNFs – in addition to their high aspect ratio and high surface area – possess unique intrinsic properties, such as abundant functional moieties and uniform dispersibility [34], that are absent in the unmodified CNTs. The functional groups of the CNFs confer hydrophilicity and strong compatibility with the polymer when it is incorporated in the polymer matrix. In addition, as opposed to CNTs, the functional moieties on the CNFs enhance the affinity of the water molecules to the nanochannels created by the nanofibers, potentially further increasing the water flux through the membrane. Also, the charged nanofibers can exert electrostatic repulsion between the contaminant particles/colloids and the membrane pore surfaces. Therefore, compared with other well-investigated nanomaterials, CNFs possess many promising attributes (i.e., high surface area and aspect ratio, abundant functional groups, good dispersibility, strong adhesion with the polymer matrix, and excellent hydrophilicity) that make them well suited for nanocomposite membrane fabrication.

In addition to the structural advantages of the CNFs compared with other advanced nanomaterials, such as CNTs and graphene oxides (GOs), the former nanomaterial can be fabricated from any natural sustainable biomass source using a facile TEMPO-mediated oxidation [32] or nitro-oxidation method [35]. In contrast, the fabrication of the latter nanomaterials requires sophisticated engineering tools with intensive pre- and post-treatment steps.

Despite the promising properties of CNF compared with other nanomaterials, the existing studies on development of CNF-polymer hybrid membranes are still very limited. This may be due to the fact that nanocellulose is typically dispersed in an aqueous phase, whereas water is a non-solvent for typical membrane polymers. In this sense, CNF must be uniformly dispersed in an organic solvent prior to its incorporation within the polymer matrix. On the other hand, the high viscosity of the CNF suspension [36], owing to the long partially-interconnected nanofibers, is a bottleneck for the incorporation of high CNF content.

In this study, we developed a facile method for the preparation of a CNF-embedded nanocomposite membrane, which consisted of cellulose acetate as the polymer matrix and the CNF as the filler to create nanochannels for preferential water flow. The hybrid membrane fabrication process was designed to be readily scalable for economic and practical applications. Cellulose acetate was chosen as the membrane matrix because of its potential van der Waals interaction with the CNF and hence better adhesion between the two phases. We believe that the similarity between the building blocks of the cellulose acetate and the CNF assists in bridging the filler-induced voids, hence increasing the water permeability without significantly sacrificing the rejection value. The impact of CNF loading was also found to be a key factor in determining the membrane performance.

## 2. Materials and Methods

### 2.1. Materials

Cellulose acetate (M.W. 100,000) supplied by Acros Organics (Somerville, NJ, USA) was used as matrix polymer in the preparation of the nanocomposite membranes. N,N-dimethylformamide (DMF, ≥99.8%) purchased from Alfa Aesar (Haverhill, MA, USA) was used as the polymer solvent without further purification. Polyvinylpyrrolidone (PVP) with an average molecular weight of 1,300,000 was supplied by Sigma Aldrich (St. Louis, MO, USA) and was used as a pore forming agent. Bovine serum albumins (BSA, fraction V, 97%) with an average molecular weight of 67 kDa and dextran with different molecular weights ranging from 4 to 5000 kDa were purchased from the Fisher Scientific and were used as received. Softwood pulp, made predominately from Loblolly Pine, was supplied by the International Paper Company (Clifton, NJ, USA) and was used for CNF production. Other chemicals used were 2,2,6,6-Tetramethyl-1-piperidinyloxy (TEMPO, 98%), sodium bromide (NaBr), and sodium hypochlorite (NaOCl, 14.5% available chlorine) and were purchased from Fisher Scientific (Hampton, NH, USA).

### 2.2. Preparation of Cellulose Nanofiber in Organic Solvent

10.0 g delignified wood pulp was dispersed in 500.0 g of deionized water. Subsequently, TEMPO oxidizing agent (0.2 g) and sodium bromide (1.0 g) were introduced into the dispersion and stirred vigorously to disperse the fibers. The pH level of the suspension was adjusted to and maintained at a value of 10.0 ± 0.2 throughout the reaction process by addition of 1 M NaOH solution. 112.0 g NaOCl was gradually added into the suspension to start the oxidation process under continuous stirring for 24 h. It was critical to maintain the pH level at ~10 to provide optimum oxidation conditions. 50 mL ethanol solution was added to quench the reaction and stirring was continued for another 30 min. The final product was separated by centrifugation at ~6000 rpm. Then, the resultant product was washed and centrifuged again. Finally, the product was placed in a dialysis bag until the conductivity of the medium was <5 μS/cm. The concentration of the bulk cellulose nanofiber (CNF) suspension was measured to be 0.3 wt %.

A predetermined amount of CNF at a specified concentration was mixed with a known quantity of DMF overnight to ensure an even distribution of CNF in a binary mixture of water/DMF. Subsequently, the water was removed from the suspension using a six-inch vacuum-assisted fractional distillation apparatus at 100 °C under vacuum. The CNF dispersed in the organic phase was then recovered and used as a stock CNF suspension for nanocomposite membrane fabrication.

### 2.3. Preparation of Nanocomposite Membranes

A predetermined amount of CNF suspension (in DMF) was obtained from the prepared stock CNF suspension and additional DMF was added into the suspension to provide the desired final CNF concentration. Three CNF concentrations in the polymer solution, 0.05, 0.1, and 0.2 wt %, were employed to make the nanocomposite membranes. Afterwards, a certain amount of polyvinylpyrrolidone (PVP), as a pore forming agent, was added to the suspension to give a final concentration of 0.5 wt % and stirred for 1 h. Then, a predetermined amount of cellulose acetate was added at several intervals over 4 h to provide a fixed cellulose acetate final concentration of 15 wt %. The gradual introduction of the cellulose acetate was essential to avoid the sudden increase in the solution viscosity and to facilitate the homogeneity of the solution. The resultant CA/CNF/PVP solution was mixed at room temperature overnight. Before its application for membrane casting, the CA/CNF/PVP solution was degassed using a hand-held vacuum pump to remove the bubbles trapped in the viscous polymer solution. Subsequently, a Gardco casting knife film applicator was used to cast the polymer solution, followed by a phase inversion process in a water bath under controlled conditions. After the nanocomposite membrane was fabricated, it was washed several times with distilled water for a few hours to ensure the removal of all the organic phase. The membrane was kept in a fresh water bath for 2 days to ensure the diffusion of the pore forming PVP to the aqueous medium. The CNF contents in the fabricated nanocomposite membranes were calculated as 0.1, 0.3, and 0.6 wt %. CA−CNF0.1, CA−CNF0.3, and CA−CNF0.6 were used to represent the membranes with different CNF contents in the nanocomposite membranes. The final thickness of the membranes was fixed at ~ 200 µm.

### 2.4. Cellulose Nanofibers (CNF) Characterization

A FEI BioTwinG2 transmission electron microscope (TEM) equipped with an AMT XR-60 CCD digital camera system (Hillsboro, OR, USA) was used to acquire images of the individual CNF. An accelerating voltage of 120 kV was applied for the TEM measurements. In the sample preparation for TEM, a 10 μL droplet of cellulose nanofiber suspension (0.01 wt %) was deposited on a carbon-coated TEM grid (Ted Pella Inc., Redding, CA, USA) and the excess liquid was absorbed by a piece of clean filter paper. Then, a small drop of 2.0% uranyl acetate negative stain was added. The uranyl acetate excess solution was subsequently removed, allowing the blotted piece to dry on the grid. 300 mL of 0.196 wt % nanocellulose water suspension was prepared and its zeta potential value at different pH (3–10) was measured at 25 °C using the ZetaProbe Analyzer (Colloidal Dynamics Inc., Ponte Vedra Beach, FL, USA). 0.1 M NaOH and HCl solutions were used for pH adjustment. The degree of oxidation (amount of carboxylate group per unit gram) of TEMPO-oxidized cellulose nanofibers was determined through the conductometric titration method. Specifically, 0.1 M hydrochloric acid solution was added to 198.5 g of 0.1 wt % CNF suspension to adjust its starting pH value to around 2.5. Under stirring, the suspension was titrated with 0.05 M standardized NaOH (Sigma-Aldrich, St. Louis, MO, USA) solution until the pH level reached 10.5 with 0.2 mL addition interval. During the titration, the conductivity was monitored after complete stabilization.

### 2.5. Membrane Characterization

The surface and cross-sectional morphologies of the nanocomposite membranes were examined by a Schottky field emission scanning electron microscope (FE-SEM) (LEO Gemini 1550, Zeiss, Oberkochen, Germany). Before SEM characterization, all the specimens were dried in a vacuum oven at 40 °C for 2 days. The membranes were cryogenically fractured in liquid nitrogen for cross-sectional imaging. All specimens were mounted on aluminum holders using a double-sided conductive tape and then sputter-coated with gold. The SEM micrographs were obtained at an accelerating voltage of 2.5 kV. The thermal behavior of the nanocomposite membranes was studied using a simultaneous thermogravimetric and differential thermal analyzer (TGA-DTA, TA Instruments Q50, New Castle, DE, USA) under a nitrogen atmosphere at a heating rate of 5 °C from 30 to 700 °C. A Perkin Elmer Spectrum One Fourier transform infrared spectrophotometer (FTIR, Waltham, MA, USA) equipped with attenuated total reflection (ATR) configuration was used to record the change in the surface functional groups of the nanocomposite membranes before and after the model protein filtration. The spectra were recorded at a resolution of 4 ${\mathrm{cm}}^{-1}$ and 64 scans per spectrum between the wavenumber range of 4000–400 ${\mathrm{cm}}^{-1}$. The surface wetting properties of the fabricated membranes were determined using a Dataphysics (OCA 15EC, Hamden, CT, USA) contact angle analyzer. Distilled water was used as the probe liquid in all the measurements. 2.5 µL water droplet was dropped on the membrane surface and the contact angle of the droplet with the membrane surface was determined using a sessile drop technique. The contact angle was measured at a minimum of 8 different locations on the membranes and the average value was reported. A zeta potential analyzer (Anton Paar, SurPASS 3, Graz, Austria) was used to study the surface charge of the membrane. The membranes with dimensions of 20 mm × 10 mm were glued onto adjustable gap cells with a gap distance of around 110–130 µm. The streaming potential measurements were carried out at a pH range of 3.5–8.5. The molecular weight cutoff (MWCO) measurements were carried out by evaluating the rejection of dextran solutes of varying molecular weights in a dead-end stirred cell (Amicon Stirred Cell, 50 mL). A Shimadzu total organic analyzer (TOC-VCPN, Kyoto, Japan) was used to measure the concentration of the dextran in the permeate.

### 2.6. Evaluation of the Nanocomposite Membrane Performance

The performance of the nanocomposite membranes was evaluated using a cross-flow system using a clear-cast acrylic Sterlitech cell with an active membrane area of 42 ${\mathrm{cm}}^{-2}$. The feed water was pumped across a given membrane specimen at a transmembrane pressure of 10 psi. Both feed and permeate water were recycled back into the reservoir. First, the pure water flux test of a given membrane was carried out for 24 h. The fluxes at different time intervals were recorded to ensure that a steady state flux was attained ($J_{0}$). Then, for fouling studies, BSA solution was added into the reservoir to provide a final BSA concentration of 100 mg/L and the filtration continued for another 4 h. The flux decline with time indicating the fouling intensity and the BSA rejection at regular interval was recorded. The BSA concentration was determined using an ultraviolet/visible spectrophotometer (UV/Vis, Thermo Scientific Genesys^TM^ 10S, Waltham, MA, USA) with a high intensity xenon lamp at a wavelength of 278 nm. The membrane permeation flux (*J*) was measured according to the following equation:

(1) J=V(A×t)

where *V* is the volume of the permeate flowing through the membrane at a certain amount of time (*t*), and *A* is the effective membrane area. The rejection of the BSA by the membranes ($R_{t}$) was determined by measuring the BSA concentration in the bulk solution ($C_{0}$) and in the permeate ($C_{t}$) as follows:

(2) $$R_{t}=\left(1-\frac{C_{t}}{C_{0}}\right)\times 100$$

## 3. Results and Discussion

### 3.1. Properties of Cellulose Nanofibers

**Figure 1:**
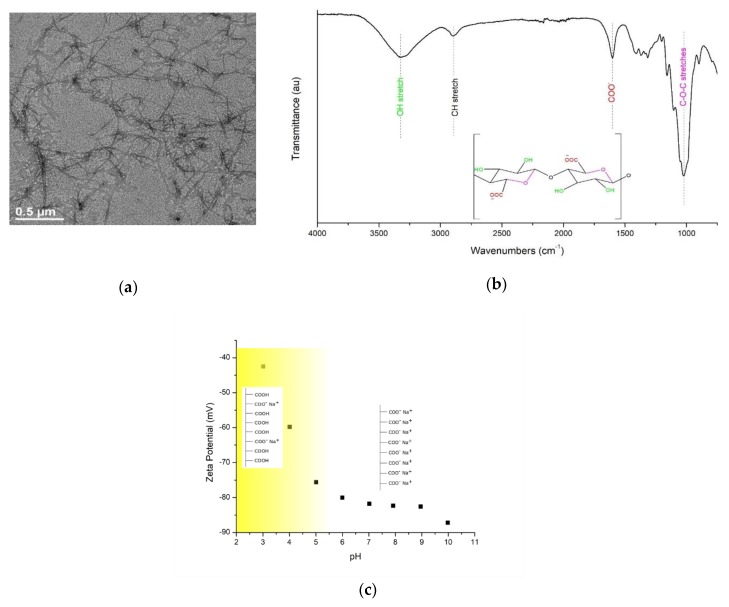
(**a**) Typical transmission electron microscopy (TEM) image of cellulose nanofibers (CNFs); (**b**) Fourier transform infrared spectrophotometer (FTIR) spectrum of a thin CNF film; (**c**) Solution-state zeta potential values of the CNF at different pH values.

Figure 1a shows TEM images of the individual CNF after the TEMPO oxidation reaction. The width of the CNF was shown to be in the range of 5 to 15 nm. Previous studies reported that the defibrillation efficiency strongly depended on the biomass source. For example, Saito et al. measured the CNF width using the TEM and atomic force microscope (AFM) and demonstrated that wood cellulose had a width of 3.6 ± 0.3 nm by TEM and 2.6 ± 0.3 nm by AFM, whereas tunicate cellulose showed a width of 13.5 ± 4.9 nm, measured by TEM [37]. Mao et al. analyzed the dimensions of the wood pulp-extracted CNF using small angle neutron scattering (SANS) and small angle X-ray scattering (SAXS) and found that the width and cross-section of the CNF were ~8 and ~2 nm, respectively [38]. Our findings are in close agreement with these measurements.

The functional groups on the CNF, specifically carboxylate groups, are essential to impart electrostatic repulsion between the cellulose nanofibers, providing a stable dispersion without aggregation. Figure 1b shows the FTIR spectrum of a dried thin film CNF. The intense and weak peaks at 3352 and 1640 cm^−1^ were attributed to the stretching and bending vibrations of the O–H groups, respectively. The peak at 1160 cm^−1^ was assigned to the C–O–C asymmetric stretching vibrations of the β-glucosidic linkages in the cellulose backbone. The band at 1730 cm^−1^ corresponded to the carboxylate groups. This peak was of particular interest to ensure the conversion of the C6 hydroxyl groups to carboxylate groups. It is recognized that cellulose nanofibers (CNFs) form strong intra- and inter-molecular hydrogen bonds due to the abundant hydroxyl moieties on the linear chains of β-1,4-glucose units. This leads to the aggregation of the cellulose chains which renders its defibrillation challenging. TEMPO-mediated oxidation method is a promising technique for the conversion of its hydroxyl groups into carboxyl and aldehyde groups mainly via regioselective nitroxyl catalytic oxidation of the C6 primary hydroxyl moieties into carboxylate groups. Therefore, the inter- and intramolecular hydrogen bonding in the CNF is significantly reduced, resulting in a less agglomerated, more dispersed suspension.

The carboxylate content of the CNF was quantified by conductometric titration of the CNF. Based on the data, the degree of oxidation of the CNF was calculated to be around 0.85 mmol/g, which is consistent with other reported literature values [39]. Such a high degree of oxidation for the CNF is expected to contribute to the negative charges on the CNF surface. As illustrated in Figure 1c, the zeta potential values of the CNF in solution-state was found to be negative throughout the pH range investigated. In particular, the zeta potential value of the CNF at a neutral pH value (pH = 7.0) was ~ −82 mV, indicating that the CNF had a remarkably-high negative surface charge. The negative charge of the CNF is desirable for water purification applications whereby the electrostatic repulsion between the negatively-charged CNF and same-charge contaminants may increase the contaminant rejection. It is noteworthy that most of the contaminants in water bodies, such as colloids, proteins, viruses, and bacteria possess partially anionic surfaces [40,41,42]. The drastic increase in zeta potential at very low pH values may result from the micro-agglomeration of the cellulose nanofibers.

### 3.2. Ultrafiltration Cellulose Acetate-Cellulose Nanofiber (CA-CNF) Nanocomposite Membranes

**Figure 2:**
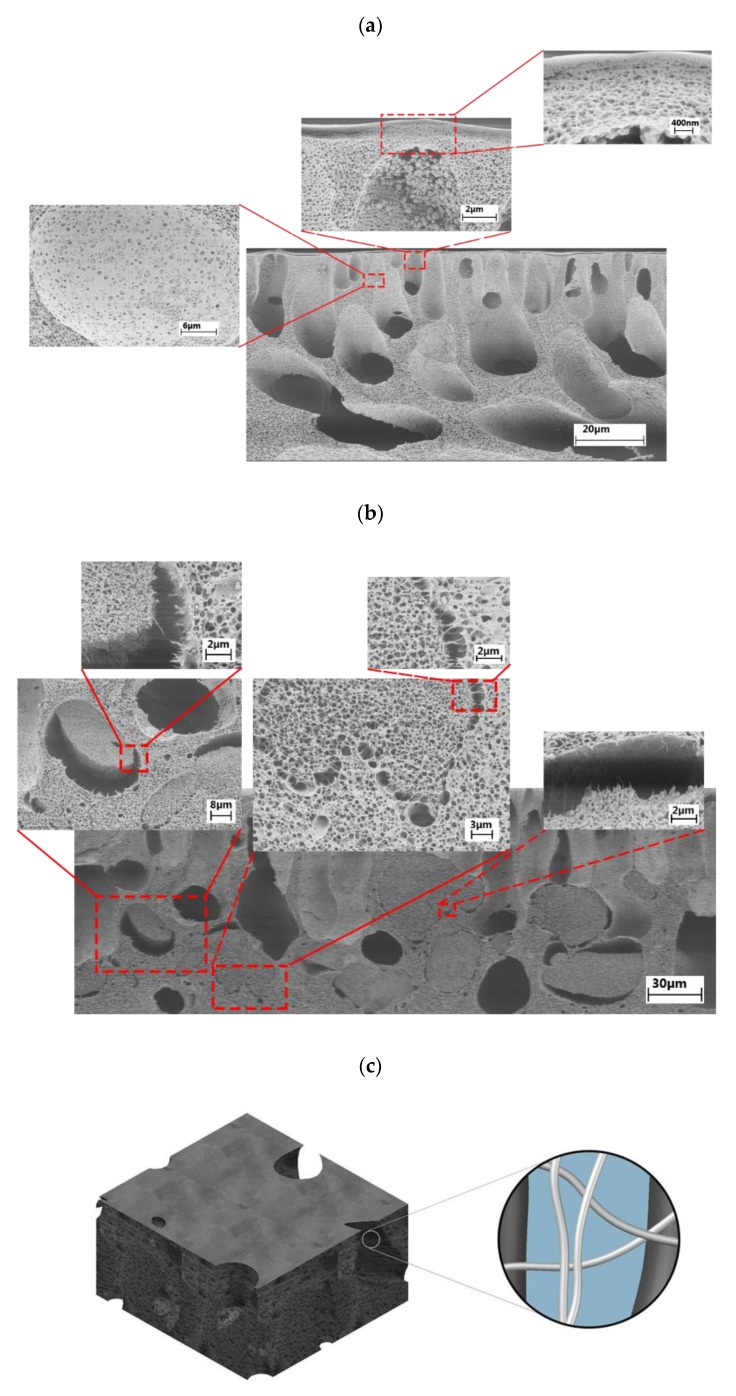
Cross section scanning electron microscopy (SEM) images of the (**a**) pristine cellulose acetate (CA) membrane and (**b**) cellulose nanofibers (CNF) embedded CA nanocomposite membrane. (**c**) Schematic representation of water directing channels in nanocomposite membranes.

The cross-sectional SEM images of the pristine cellulose acetate (CA) and a typical CNF-embedded CA nanocomposite membrane are illustrated in Figure 2a,b, respectively. The pristine CA membrane exhibited a very dense barrier layer structure with a barrier layer thickness of ~100–200 nm, where the major fraction of the contaminant rejection occurred. Below the barrier layer, the pristine membrane showed a maze-like porous structure. The water permeate needed to penetrate through these pores until it reached the “finger-like” macrovoids. However, it can be clearly observed in Figure 2a that some of the pores were completely clogged by two distinct phenomena. Firstly, the pores were formed in a regular pattern, but they were not interconnected to the other pores, and thus, the transport phenomenon through the membrane was not efficiently accomplished. Secondly, some of the pore forming agent (PVP) remained in the polymer matrix as globules and could not be removed probably due to the very small pore sizes, which hindered the diffusion of the water into the pores and macrovoids during the washing process. In addition, the macrovoids were interconnected through the walls which obviously had small pores, analogous to the other parts of the membrane, creating another barrier to transport. In other words, the diffusion of the water molecules from one macrovoid to another was hindered by the less-porous walls between these macrovoids.

The porous structure of the CNF-embedded nanocomposite membrane, on the other hand, was remarkably different from the pristine membrane. It was found that the nanocomposite membrane was comprised of two distinct phases. One phase was noticeably denser than the other phase. We postulate that these phases were CNF-rich and CNF-poor domains and thus, possessed different shrinkage behavior, leading to the formation of two distinct regions. The CNF-induced macrovoids, however, were bridged by CNF, interconnecting these water transport channels to each other, thereby maintaining the structural integrity of the membrane. Furthermore, the two CNF-rich and CNF-poor domains exhibited different solvent-nonsolvent exchange rates, probably due to the difference in the composition, hydrophilicity, surface tension, and viscosity of the solutions, resulting in distinct differences in the pore density in the two domains. In addition, the globules of the PVP were not noticeable in the nanocomposite membranes, indicating that the water diffused well into the pores and macrovoids during the washing process, removed the PVP, and opened up the pores. This was due to the fact that water could easily diffuse into and leave the polymer matrix without significant membrane resistance. In other words, these channels reduced the intrinsic membrane resistance for water transport, and thus, could potentially enhance the water permeance. In contrast to high pressure reverse osmosis processes, where the presence of macrovoids is unsatisfactory and results in membrane collapse, the presence of macrovoids is desirable in ultrafiltration membranes in order to increase their flux by reducing the membrane resistance [43,44].

It is known that the relative rate of the solvent–nonsolvent medium interchange during the gelation process is a key factor in determining the properties of the membrane [44,45]. In the pristine cellulose acetate membrane, the instantaneous aqueous–organic medium interchange resulted in the formation of a thin but dense barrier layer, separating the nonsolvent bath from the non-gelled polymer beneath the barrier layer. Further gelation of the polymer was accomplished by the bilateral diffusion of the solvent-nonsolvent media through the barrier layer. However, since the barrier layer was too dense, the gelation of the polymer beneath the barrier layer was a very slow process, leading to the formation of a finger-like structure. The incorporation of the CNF into the polymer solution resulted in the formation of CNF-rich and CNF-poor nuclei that could have different solvent-nonsolvent interchange rates and shrinkage intensities. When the aqueous phase was exchanged with the organic phase in the CNF-embedded casting solution, the polymer shrank during the membrane gelation [44]; however, the CNFs, in suspension form, maintained their robust structures and no shrinkage occurred. This led to the formation of two distinct domains separated with cavities, but well-interconnected with robust cellulose nanofibers (CNFs). It is postulated that the similarity between the building blocks of the matrix polymer and the CNF induced the bridging effect between the two domains (the polymer matrix and the filler). The schematic modelling of the water-directing nanochannel formation is presented in Figure 2c.

**Figure 3:**
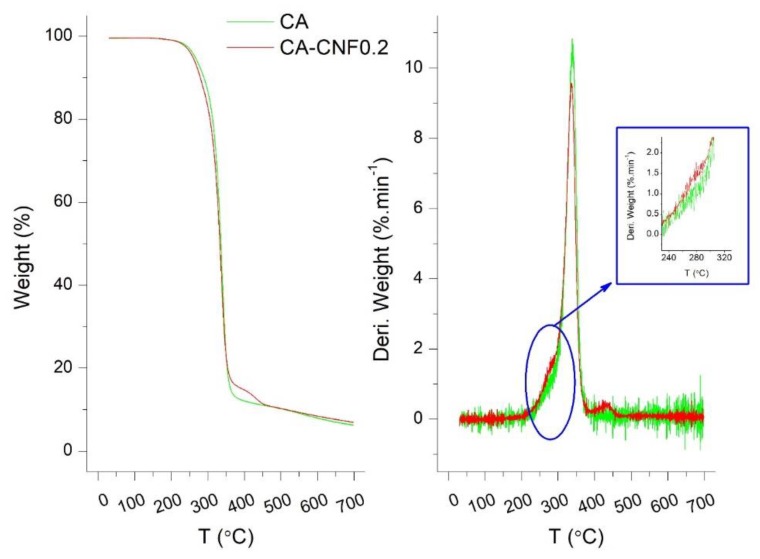
Thermogravimetric and differential thermal analyzer (DG-DTA) curve for the pristine and cellulose acetate–carbon nanofibers (CA−CNF)0.6 nanocomposite membrane.

The thermal behavior of the pristine CA membrane and the CA−CNF0.6 nanocomposite membrane were investigated by thermogravimetric analysis (TGA) coupled with differential thermal analysis (DTA). As illustrated in Figure 3, the thermal behavior of the membranes followed a three-step degradation process. There was a mass loss of ~2% starting from room temperature up to 150 °C which is due to the elimination of moisture. Then, a sharp weight decline was observed in the pyrolytic temperature range of 231–390 °C. The main degradation, centered at 338 °C, was assigned to the pyrolytic degradation of the 1,4-β-glycosidic linkages followed by the degradation of C–C, C–O, and C–H bonds in the cyclic glucose units [46,47]. The shoulder at 218–295 °C in the DTA curve represented the early degradation of the less thermally stable functional groups, such as the hydroxyl, carboxylate, and dialdehyde groups. Kannan et al. reported an onset degradation temperature of 290 °C in carbon nanotube (CNT)–Nafion membranes and assigned it to the degradation of sulfonic acid groups in the CNT side chains [48]. As mentioned earlier, cellulose acetate is only partially (40–50%) acetylated, and thus, there are still a lot of hydroxyl functional groups on its surface. Given the more abundant functional groups on the CNF, this shoulder was slightly more prominent in the CA-CNF nanocomposite membrane. However, since CNF constitutes only a small fraction of the nanocomposite membrane (0.6 wt % in this case), the change was only minimally observed. It is expected that if higher concentration of CNF were present in the nanocomposite membrane structure, the difference would be more significant.

**Figure 4:**
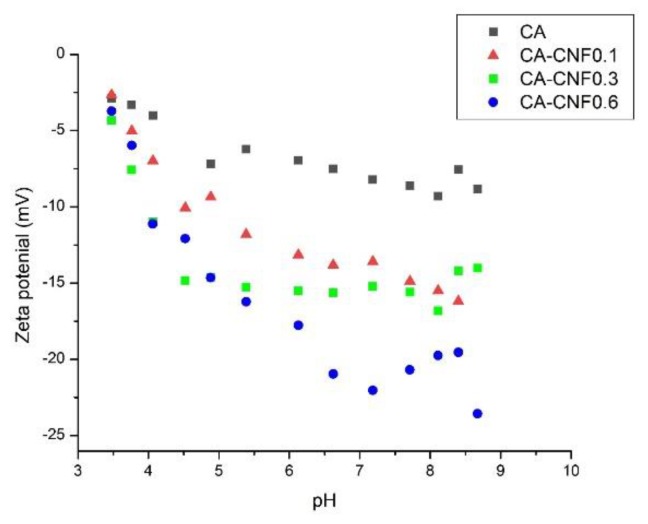
Zeta potential values of the pristine and nanocomposite membranes.

The zeta potential values of the pristine and nanocomposite membranes as a function of pH are presented in Figure 4. It was found that the pristine cellulose acetate membrane had a slightly negative surface charge. Practically, only 40–50% of the primary hydroxyl groups on the cellulose surface were converted to acetyl during the acetylation reaction [49] and therefore, some hydroxyl groups still remained unreacted on the cellulose acetate, contributing to the slightly low negative charge of the CA membrane. The change in the zeta potential values of the CA membrane by varying the pH of the electrolyte was very minor, indicative of the low charge density of the pristine CA membrane.

The nanocomposite membranes, on the other hand, were more negatively-charged throughout the pH range examined. Furthermore, as the CNF content in the polymer matrix increased, the membrane possessed more negative surface charges. For example, at a fixed pH value of 7.1, the zeta potential value for the CA, CA−CNF0.1, CA−CNF0.3, and CA−CNF0.6 were −8.2, −13.6, −15.2, and −22.0 mV, respectively. The more negative zeta potential values of the nanocomposite membranes with increasing CNF content were related to the increasing carboxylate content on the membrane surface, resulting in more negative surface charge. Also, since these carboxylate groups can easily be protonated at lower pH and deprotonated at higher pH, the zeta potential values of the nanocomposite membranes exhibited more pH dependence than the pristine CA membrane. More negative zeta potential values of membranes are desirable in water filtration, as it may assist to decrease the fouling tendency of the membranes as a result of the electrostatic repulsion between the membrane surface and the foulants in water. Also, a highly negative zeta potential may enhance contaminant rejection by an electrostatic repulsion mechanism.

**Figure 5:**
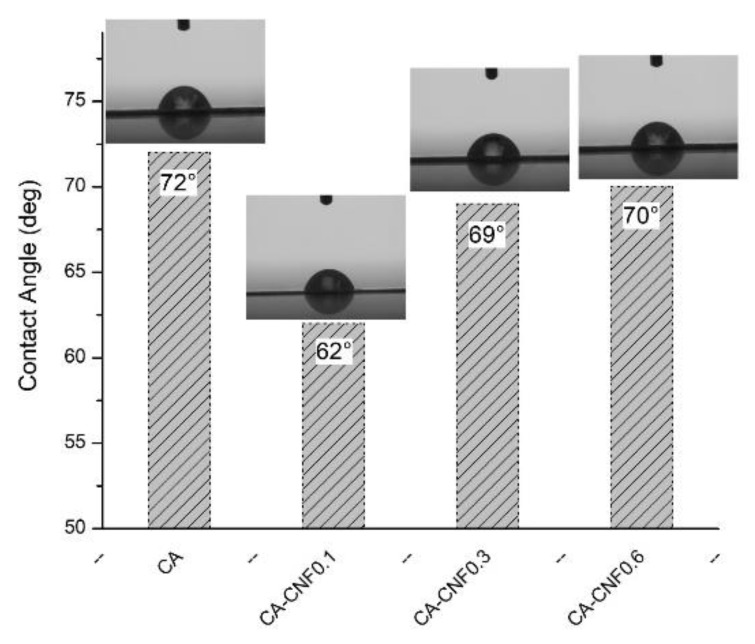
Contact angle values of the pristine and nanocomposite membranes.

The contact angles of the pristine and nanocomposite membranes are shown in Figure 5. The pristine membrane exhibited a contact angle of ~72°. When 0.1 wt % CNF was embedded in the membrane matrix, the contact angle of the CA−CNF0.1 membrane decreased to ~62°. Such a decrease in contact angle demonstrated a slight hydrophilization of the membrane as a result of introduction of hydrophilic CNF. Generally, the hydrophilicity of the CNF results from the hydroxyl and carboxylate moieties on the CNF surface. Therefore, when CNF was introduced to the membrane matrix, some of the CNF was incorporated into the membrane surface, exposing functional moieties (as demonstrated earlier by zeta potential measurements), rendering slightly greater hydrophilicity to the membrane. The reduction in the contact angle was relatively low, however, probably because most of the CNF was embedded in the membrane matrix rather than on the surface. The introduction of larger amounts of hydrophilic CNF (i.e., CA−CNF0.3 and CA−CNF0.6) increased the contact angles of the membranes to ~70°. This observation may be related to the increase in the surface roughness of the membranes. When the content of CNF incorporated into the membrane surface was high, the water affinity enhanced due to the introduction of higher amounts of functional moieties (as demonstrated earlier by zeta potential measurements), but, as opposed to polymers (such as CA) which can develop very smooth surfaces, the robust CNF created a rough surface that increased the contact angle of the membranes.

### 3.3. Ultrafiltration Performance of the Nanocomposite Membranes

**Figure 6:**
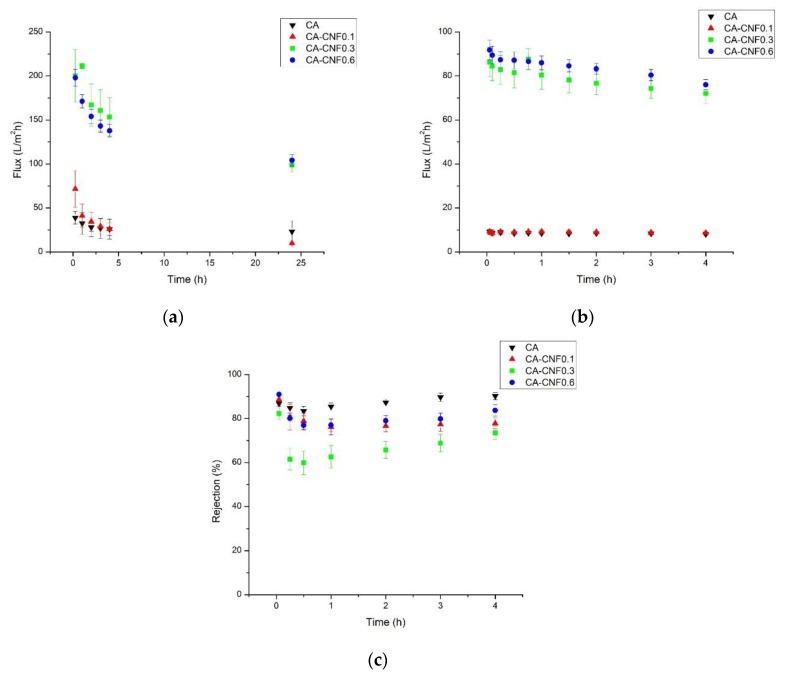
(**a**) Pure water flux, (**b**) fouling tendency, and (**c**) bovine serum albumins (BSA) rejection of the pristine and nanocomposite membranes.

Figure 6a shows the pure water flux of the pristine and CNF-embedded nanocellulose membranes. All the membranes exhibited a decrease in water flux with time. This reduction is known to be due to the compaction of the membrane under the applied transmembrane pressure. The pristine cellulose acetate membrane exhibited a pure water flux of ~15 $L\cdot m^{-2}\cdot h^{-1}$ after the compaction of the membrane was completed. Incorporation of 0.1 wt % CNF into the membrane did not change the water permeability of the membrane, probably due to the very low content of the CNF. Interestingly, a noticeable change was found when 0.3 wt % CNF was incorporated into the membrane matrix, where the water flux through the CA−CNF0.3 (102 $L\cdot m^{-2}\cdot h^{-1}$) was seven times higher than the pristine CA membrane. This increase in water flux suggests that intertwined nanochannels were formed which enabled the transport water through the membrane. Increasing the CNF concentration to 0.6 wt % in the membrane matrix did not further enhance the water permeability. In other words, incorporation of very small amounts of CNF (e.g., 0.1 wt %) resulted in an irregular sporadic arrangement of the CNF-induced nanochannels. These nanochannels were isolated and patchy in the polymer matrix due to the very low CNF content and thus, no interconnection between these nanochannels existed. This accounted for the similar water flux between the pristine membrane and the CA membrane embedded with 0.1 wt % CNF. When higher CNF contents were introduced, more nanochannels were formed and also the created nanochannels were interconnected, contributing to the greater transport of water through the membrane via the web-like structure of the nanochannels.

The fouling tendency of the membranes, illustrated in Figure 6b, showed that the membranes with higher CNF contents, i.e., CA−CNF0.3 and CA−CNF0.6, had slightly higher fouling tendency than the membranes with no or very low CNF content. CA, CA−CNF0.1, CA−CNF0.3, and CA−CNF0.6 with pure water fluxes of 9.2 $L\cdot m^{-2}\cdot h^{-1}$, 9.3 $L\cdot m^{-2}\cdot h^{-1}$, 86.3 $L\cdot m^{-2}\cdot h^{-1}$, and 91.8 $L\cdot m^{-2}\cdot h^{-1}$, respectively, exhibited flux declines of 12.5%, 6.5%, 16.6%, and 17.2%, respectively. In general, there was a relationship between the membrane flux and membrane fouling, where higher membrane flux resulted in higher membrane fouling. Xiao et al. also found that membrane properties, in addition to the foulant characteristics, play an important role in fouling propensity of the membranes [50]. They found that, for similar foulant type and membrane material, as the membrane flux increased, the fouling intensity increased. Our results corroborate their findings. Notably, although pristine CA membrane and CA−CNF0.1 had similar water fluxes, the nanocomposite membrane showed reduced fouling intensity. This was probably due to the slightly higher negative charge on the nanocomposite membrane surface as a result of the carboxylate groups of the embedded CNF, as demonstrated earlier by zeta potential measurements. It is notable that although nanocomposite membranes with high CNF contents (i.e., CA−CNF0.3, and CA−CNF0.6) exhibited higher fouling, their permeabilities after fouling were around nine times higher than the CA membrane.

The rejection of BSA by the pristine CA membrane was shown to be 84–90%, as depicted in Figure 6c. The protein rejection of the CA−CNF0.1 nanocomposite membrane was comparable to the pristine membrane. When the CNF content in the polymer matrix increased to 0.3 wt %, the BSA rejection dropped to 60–73%. This trend is very consistent with the change in the flux by the introduction of the CNF. The low CNF membrane (CA−CNF0.1) behaved similarly to the pristine membrane, because the CNF content was low so that it did not have a consequential effect on the membrane properties, including BSA rejection. However, when the CNF content increased to 0.3 wt % and the flux significantly increased, the BSA molecules could penetrate through the comparatively large nanochannels that were induced by the CNF, resulting in lower rejection. Several authors previously reported a trade-off between the permeability and selectivity of the membranes, where increasing the permeability led to decreasing the selectivity [51]. When the CNF content was increased to 0.6 wt %, the rejection increased to 80–85%. In other words, when the CNF content was as high as 0.6 wt %, not only was the flux enhanced by several orders of magnitude, but also the rejection was not sacrificed. The increase in the rejection efficiency of the membrane by increasing the CNF-loading was attributed to the bridging effect of the additional CNF. When very high amount of CNF was added to the polymer matrix, the extra CNF – that did not participate in the nanochannel formation – bridged the CNF-induced voids between the fibers and the polymer matrix and enhanced the BSA rejection. The bridging effect of the CNF in the filler-induced channels are clearly illustrated in the SEM micrographs. In addition, the increase in the negative charge of the nanochannels by CNF over-loading could also positively impact the rejection efficiency of the membrane. This explanation is consistent with the zeta potential results (Figure 4).

## 4. Conclusions

In this study, we investigated the change in the permeability and selectivity of a cellulose acetate membrane by incorporating cellulose nanofiber (CNF) into its matrix. The results demonstrated that the incorporation of low amounts of CNF (0.1 wt %) did not significantly change the permeability or selectivity of the membranes. Incorporation of very small amounts of CNF (e.g., 0.1 wt %) resulted in an irregular sporadic arrangement of the CNF, which created isolated and patchy nanochannels in the polymer matrix. Thus, no interconnection between these nanochannels existed, which accounted for the similar membrane performance between the pristine membrane and the CA membrane embedded with very low amount of the CNF. However, significant changes in permeability were noticed when further CNF (0.3 wt %) was added to the polymer matrix, whereby the permeability of the membrane was increased several times to ~102 LMH, though the BSA selectivity of the membrane decreased slightly from 90% to 73%. This was due to the interconnection of the nanochannels and easier transport of the water molecules through the membrane. Also, some of the BSA molecules could escape through these nanochannels, resulting in a lower membrane selectivity. Interestingly, when the CNF content was increased to 0.6 wt %, the flux did not further increase, but the selectivity was significantly increased to 85%. This was related to the electrostatic repulsion between the membrane overloaded with CNF and the BSA, resulting in higher BSA rejection. Overall, incorporation of CNF in the membrane matrix significantly enhanced the membrane permeability without sacrificing the selectivity and therefore, CNF can be a potential candidate as a commercial additive in nanocomposite membrane fabrication.
